# Warning and green labels versus Nutri-Score: visual attention and product evaluation in a binational eye-tracking study

**DOI:** 10.3389/fnut.2026.1826542

**Published:** 2026-08-27

**Authors:** Gerrit Hummel, Eyal Ert, Lena Kurzrock, Ilan Hadad, Franziska Lang, Or Avishai Ofer, Aron M. Troen, Nanette Stroebele-Benschop

**Affiliations:** 1 Institute for Nutritional Medicine, Department Nutritional Psychology, University of Hohenheim, Stuttgart, Germany; 2 Institute of Environmental Sciences, Department Environmental Economics & Management, Robert H. Smith Faculty of Agriculture, Food and Environment, The Hebrew University of Jerusalem, Rehovot, Israel; 3 Institute of Biochemistry, Food Science and Nutrition, Robert H. Smith Faculty of Agriculture, Food and Environment, The Hebrew University of Jerusalem, Rehovot, Israel

**Keywords:** eye-tracking, food choice, front-of-pack nutrition labeling, Nutri-Score, visual attention, warning labels

## Abstract

Front-of-package labeling (FOPL) is intended to improve consumer awareness of nutritional quality and encourage healthier choices. In 2020, Germany and Israel implemented distinct FOPL systems: Israel introduced mandatory red warning labels with a voluntary green positive label for healthy foods, while Germany adopted the Nutri-Score. This study compared visual attention to these two FOPL schemes and their impact on product perception in a binational context. We conducted a comparative eye-tracking study with 223 participants (112 in Germany, 111 in Israel). Each participant viewed food package images, each displaying either a Nutri-Score or a red warning or green label, while their eye-movements were recorded. Then, participants rated perceived product healthfulness, expected taste, and purchase intention and completed questionnaires on eating habits, nutritional knowledge, and label understanding and usage. Outcomes were analyzed with mixed-effects models and *t*-tests. Label system and familiarity influenced visual attention and consumer evaluations. Red warning labels draw more attention when novel, while familiar labels are noticed faster but scrutinized less. Perceived healthfulness of poorer nutritional products was higher under Nutri-Score compared to explicit warnings. Participants also rated warning labels as easier to understand and more helpful for making healthy choices than Nutri-Score labels in both countries. These findings underscore the importance of empirical evaluation of both attention-catching ability and user comprehension in designing effective FOPL interventions.

## 1 Introduction

Obesity and overweight have evolved into a global epidemic, affecting millions across diverse demographics. Reports from the World Health Organization (WHO) show that around one in eight people worldwide are considered obese (1, 2). The WHO strongly encourages governments to develop strategies and measures to raise awareness of nutritional composition of food and promote healthier dietary patterns (3, 4). A range of policy measures, such as fiscal policies (e.g., taxes) and effective labeling policies, are considered promising approaches for improving nutrition and preventing a further increase in obesity (5). Front-of-package labeling (FOPL) is a strategy adopted by many countries worldwide to encourage healthier food choices by consumers (6), and to encourage food producers and sellers to improve the health-profile of their products. The present study contributes to this body of literature by examining how different FOPLs influence visual attention toward front of packs and how they affect product evaluations in two non-representative adult samples.

Front-of-pack nutrition labels provide simplified nutrition information on the front of food packages, using accessible visual cues – such as color coding, symbols, shapes, grading scales, or signal wording – to help consumers interpret a product’s nutritional quality at a glance and guide food choices without directly restricting choice or taxing products (7). Various FOPL schemes have been introduced worldwide, including interpretive and directive systems such as nutrient-specific warning labels and summary evaluative labels such as Nutri-Score [e.g., (6, 8)]. However, there is no consensus on the optimal FOPL design, and different stakeholders continue to advocate for different schemes.

These schemes are not merely graphically distinct; they embody different assumptions about how labels influence consumer behavior and reflect different policy priorities. Nutri-Score provides a summary, graded evaluation of the overall nutritional quality of a product, thereby facilitating rapid comparison among products within or across categories. In contrast, high-in warning labels identify products that exceed thresholds for nutrients of public health concern, such as sugar, sodium, saturated fat, or energy, thereby increasing the salience of risk and directing consumers away from products carrying warnings. Thus, although both systems are intended to encourage healthier food choices, they may operate through different cognitive and behavioral mechanisms and may differ in their effectiveness across populations, product categories, and purchasing contexts.

The WHO has identified FOPL as an important policy tool for promoting healthier diets and recommends that governments develop, implement, and evaluate systems appropriate to their populations. In the EU, the European Commission committed under the Farm to Fork Strategy to propose a harmonized mandatory FOPL, but disagreement among Member States, industry actors, and public-health stakeholders has delayed a final decision. This unresolved European debate reflects a broader global uncertainty over FOPL design, particularly over whether policy should prioritize summary nutritional guidance, nutrient-specific risk warnings, consumer acceptability, reformulation incentives, or harmonization across markets.

**FIGURE 1:**
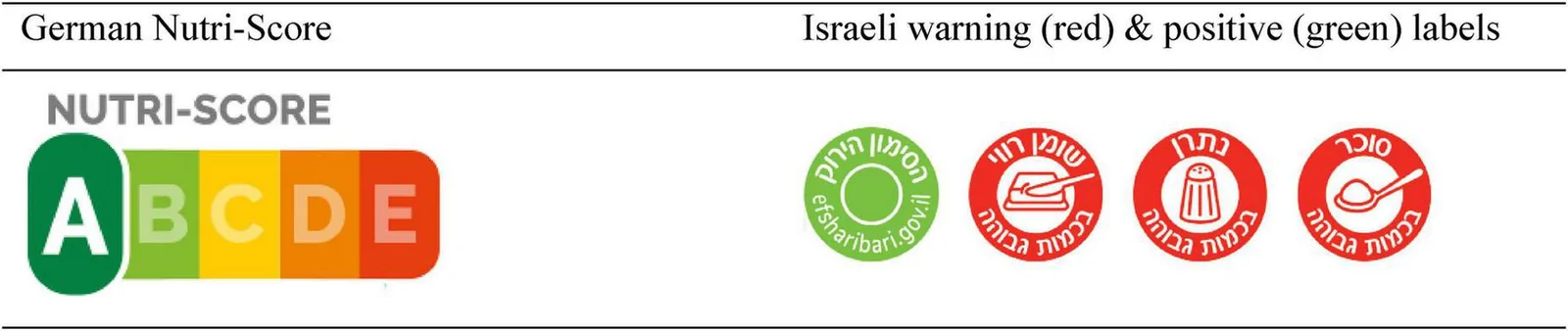
German and Israeli FOPL design. Left: German Nutri-Score (example shows an “A”). Nutri-Score-A, Public Domain via Wikimedia Commons. Right: Israeli label system consisting of red warning labels (“High in” saturated fat, sodium, sugar) and green positive label (“The green label”). Israeli red warning labels, saturated fat, sodium, sugar and green positive label, Public Domain via Wikimedia Commons.

One of the most prominent directive label is the Nutri-Score, which ranks foods based on overall nutritional quality using a five-color scale from A (green, healthiest) to E (red, least healthy). The Nutri-Score calculation is based on a formula that balances positive components (e.g., fiber, protein, fruit and vegetable content) against negative attributes (total energy, sugar, salt, and saturated fat) to produce a single nutrition score (9). It allows consumers to compare the nutritional profiles of products within the same category and is intended to promote healthier choices (10, 11). The Nutri-Score has been implemented in several European countries, including Germany, where it was introduced in 2020 on a voluntary basis. Another prominent FOPL scheme are warning labels, which have been implemented by most South American countries as mandatory black hexagon symbols. In Europe, the only existing label system consisting of warning labels is Israel’s red warning circles (implemented in 2020) that mark products high in sugar, sodium, and saturated fat to discourage their consumption (12). Interestingly, the Israeli FOPL implementation also included a voluntary green “positive” label to promote certain unprocessed foods deemed healthy by the Ministry of Health. This combination of mandatory warning labels and a voluntary positive label is unique to Israel. Both types of labels are used together in the food environment in Israel, thus forming the Israeli FOPL system. Nutri-Score and warnings are both directive interpretive labels, yet they differ in their approach to informing the consumer. The Nutri-Score is described as a tool to facilitate comparative processing (13). In contrast, the Israeli warning label explicitly alerts consumers to high levels of specific nutrients (sugar, salt, and/or saturated fat) in a product, aiming to discourage purchase and consumption of any product bearing these “High-in” warnings (14). Both FOPL designs use unique color codes and warning labels also use signal words (see Figure 1) to suggest actions to consumers.

The introduction of the various FOPL systems entails the evaluation of their effectiveness. Grunert & Wills described a theoretical framework that summarizes various steps involved in the use of FOPLs that are also critical for the evaluation of the effectiveness of FOPLs (15). The framework includes perception, understanding, attitudes, and effects on food choices (16).

Evidence for the effectiveness of the Nutri-Score in improving consumers’ understanding of nutrition and ability to discriminate the nutritional quality of foods is promising (17, 18). However, its impact on health perceptions and purchasing behavior is less clear, with mixed results reported (13, 19–21). Evidence for warning labels also supports their effectiveness, yet research to date has focused mostly on South American implementations of the black hexagon design. The Israeli design (red/green circles) has not been studied in depth, aside from a few self-reported surveys which suggest that consumers understand the labels and intend to purchase fewer red-labeled and more non-labeled or green-labeled products (22, 23). Yet the intended influence of the Israeli FOPL system on consumers have never been studied experimentally. Taken together, the evidence on both labeling systems reveals a significant research gap regarding their effectiveness in capturing consumer’s attention, and influencing perceptions on product healthiness. In addition, the research on the effect of front-of-pack-labeling (FOPL) on consumers has not yet addressed potential long-term influences, such as familiarity. Consumers’ familiarity with labels may increase their effectiveness through learning but may also reduce attentiveness due to habituation. The comparison between German and Israeli consumers’ responses to both labeling systems provides an opportunity to examine the role of familiarity in shaping attention. Specifically, it allows us to compare each sample’s response to its own FOPL (familiar) with its response to the FOPL implemented in the other country (novel).

Prior comparative investigations have largely relied on surveys and have compared several FOPL systems [e.g., (16, 18, 24–26)]. Hammond et al. (25) showed in a cross-country comparative study that simpler label designs (e.g., warning labels in Chile) were better understood, and that label use was highest when the label was mandatory and already implemented in the respondents’ country. This interesting observation influenced our hypothesis generation, as it suggests that familiarity with the FOPL affects its perception. Egnell et al. (16, 24) carried out similar and comparable cross-country studies in Europe. In their first study, Egnell et al. (24) showed that the Nutri-Score performed best while warning labels and reference intake label performed worst. One argument supporting this finding was the salience of the color coded FOPLs compared to the black warning labels or the light blue reference intake labels used in the study. The learned associations for red “stop” and for green “go” (27) may be influencing the information processing in different stages (16, 24). First at earlier stages of visual attention – known as initial orientation – and later by interpreting and aiding understanding (24), which can be observed as maintained attention. Similar to Egnell et al. (16) König et al. (28) showed in a cross-country study that Nutri-Score is more effective than black warning labels in identifying the healthier choice. Until today, studies comparing different FOPLs [e.g., (16, 18, 24, 28)] have focused on black warning labels instead of the Israeli warning labels. And yet, it is particularly the Israeli label system, which consists exclusively of the signal colors red and green, that may convey a “stop” or “go” message even more clearly and effectively than the Nutri-Score. A direct comparison of the Nutri-Score with the Israeli label system would therefore provide a useful empirical setting for testing the “color salience hypothesis” (16, 18, 28). In order to investigate this assumption using a broader set of methods, we follow the suggestion made by Grunert and Wills (15).

Grunert and Wills (15) pointed out the need for studies that go beyond self-reported information and are supplemented by eye-tracking experiments in the lab to measure and incorporate conscious and subconscious perceptions. Visual attention has a significant influence on perception (29) and eye-tracking is an objective, instrument-based method that is part of the standard repertoire for assessing the perception of FOPLs nowadays [see e.g., (30–33)]. Processes of bottom-up and top-down control of visual attention (29, 34) can be understood as measurable indicators of conscious and subconscious perception. Bottom-up processes are defined as stimulus driven attention which is mostly influenced by the visual salience of the stimulus itself (29) or the ‘conspicuity of a visual object relative to its surroundings’ [(34), p.188] such as the labels’ location, its color, the size or the shapes (35). In contrast, top-down control is for example driven by individual goals and preferences, cognitive load, involvement and mood (34) or regarding food labels for example based on knowledge, use or understanding of food labels (35). Eye-tracking experiments confirm that color has a significant influence on the perception and selection of food (36, 37) and the perception of FOPLs (35).

In summary, we derived the following assumptions: First, we expect that the explicit “High in” warnings in combination with the red signaling colors will have a strong impact on consumers (38), leading to more negative perceptions of products’ healthiness and lower purchase likelihood compared to an equivalent Nutri-Score (28). The expressive wording is complemented by the salient signal-colored scheme of the Israeli labeling system (4). Eye-tracking studies have demonstrated that salient colors attract more visual attention (36, 37, 39). We therefore formulate these assumptions as *warning hypotheses* and relate them to the relevant areas of visual attention and subjective assessment of health, expected taste, and purchase likelihood.

H1a) *Products with Israeli nutrition labels receive more visual attention than products with Nutri-Score*.

H1b) *The presence of Israeli nutrition labels, as compared to the Nutri-Score, has a negative impact on the subjective assessments of products.*

Second, we posited that familiarity with a label might play a crucial role (25). People who were exposed to a particular FOPL (e.g., Israelis with warning labels, Germans with Nutri-Score) may have integrated it into their everyday choices, whereas an unfamiliar label might draw more scrutiny or curiosity (40, 41). Thus, it is hypothesized that participants will evaluate products with familiar nutrition labels differently regarding their expected taste, healthiness, and their likelihood to purchase the products.

H2a) *Visual attention differs between familiar and unfamiliar labels.*

H2b) Individuals evaluate products with familiar and unfamiliar nutrition labels differently.

**FIGURE 2:**
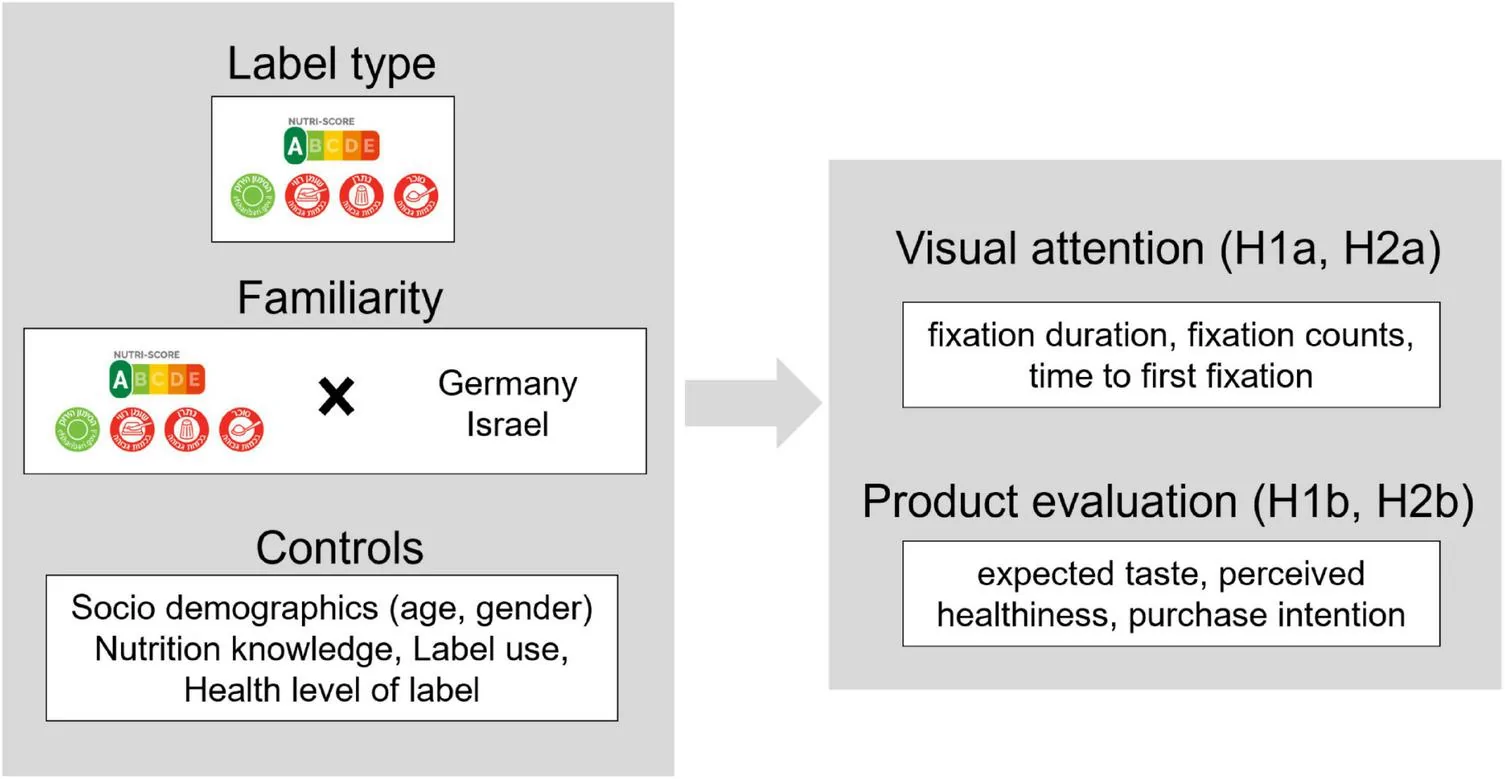
Conceptual framework. Nutri-Score-A, Public Domain via Wikimedia Commons and Israeli red warning labels, saturated fat, sodium, sugar and green positive label, Public Domain via Wikimedia Commons.

All concepts, variables and the proposed causal relationships among them are summarized and visualized in the conceptual framework of our study (Figure 2).

## 2 Methods

The study is a pre-registered (AsPredicted #137552) comparative experiment conducted in two countries (Israel and Germany) involving two different FOPL systems (the Israeli label system including red warning label and green label and the German Nutri-Score). The study was carried out in accordance with the Declaration of Helsinki and was approved by the ethics committees of the participating universities.

### 2.1 Sample and participant recruitment

This study used a convenience sample of 241 participants, recruited between May and August 2023 on and around the university campuses in each country through direct personal contact, emails, and social media, to obtain samples that were as comparable as possible across countries. To achieve sufficient statistical power, we aimed to recruit at least 100 participants per country (for a total sample of at least 200). Inclusion criteria were: age ≥18 years; being a student, employee, or visitor of the university; and fluency in the local official language (Hebrew in Israel, German in Germany) to follow instructions, answer questions, and provide informed consent. Individuals with specialized nutrition knowledge (e.g., current or former nutrition science students, dietitians, or nutrition professionals) were excluded to avoid expert bias. For eye-tracking feasibility, people who wore hard contact lenses or varifocals, or had eye diseases, were also excluded. All participants gave written informed consent before participation.

### 2.2 Procedure

**FIGURE 3:**
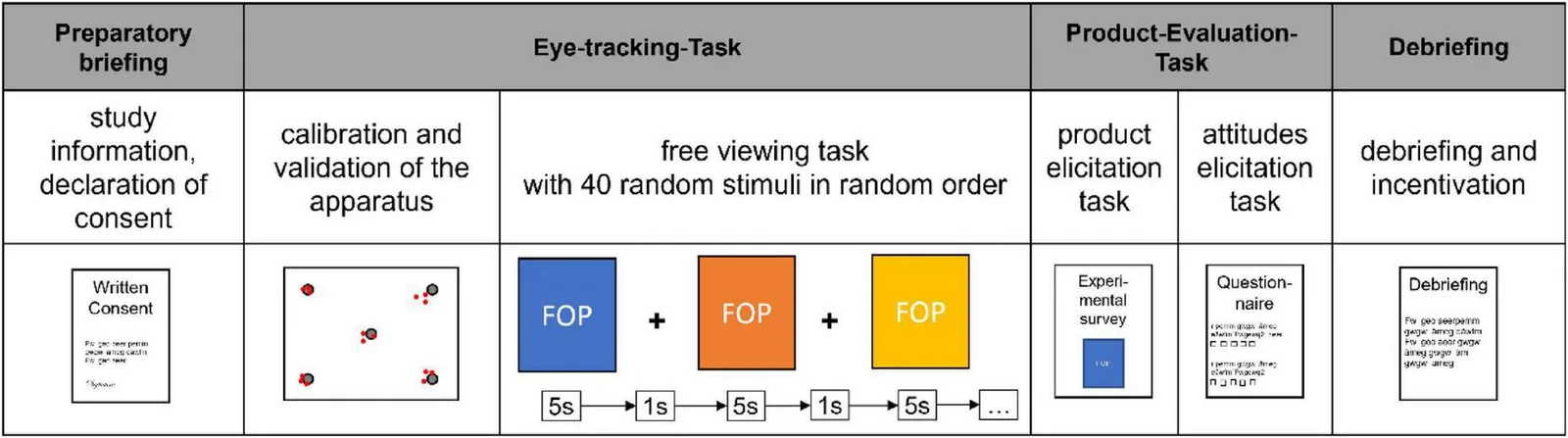
Study procedure.

The experiment consisted of two parts. The first part was an eye-tracking task to record visual attention to front-of-pack nutrition labels. The second part was a survey and product evaluation task including various questionnaires (see Figure 3). The procedure was identical in both countries, with materials provided in Hebrew in Israel and in German in Germany.

After screening for eligibility and obtaining consent, participants were seated in front of a monitor equipped with an eye-tracking device. After individual calibration and validation of the eye-tracker the participant was shown a randomized sequence of 40 front-of-package stimuli (food package images) for 5 s each. During each 5-s display, the participant could freely view the product image. To reduce potential uncontrolled biases in initial fixations, such as left-right biases that might arise from differences in reading direction, a 1-s fixation cross appeared between stimuli to re-center gaze. While participants viewed the packages with nutrition labels, the eye-tracker recorded their eye movements.

Upon completion of the eye-tracking sequence, participants proceeded to the experimental survey in which they rated selected food items (cashews, cracker, cornflakes, and protein bar). Each of these items was once presented as carrying a Nutri-Score and once as carrying a warning or a green label (Supplementary Table 1). Participants rated each version of these products on expected taste, perceived healthiness, and likelihood of purchase. Following these ratings, participants answered questionnaires regarding their eating habits, nutrition knowledge, familiarity and usage of FOPL, and other socio-demographic characteristics. Finally, each participant was debriefed about the purpose of the study and received a small compensation (a supermarket voucher worth 50 shekels in Israel or €5 in Germany).

### 2.3 Eye-tracking apparatus and measures

To ensure comparable technical conditions in both countries, we used similar hardware and identical software versions for eye-tracking. In Germany, visual attention was recorded with a Tobii Pro Fusion eye-tracker (Tobii AB, Sweden), and in Israel with a Tobii Pro Spectrum; both devices were run at 60 Hz. The same software (Tobii Pro Lab version 1.217.49450/Tobii Pro Manager version 2.5.0) was used at both sites, enabling us to work with a shared project and identical settings. Stimuli were presented on a 1920 × 1080 resolution screen, and participants sat ∼60 cm from the monitor in an isolated booth. Each participant’s eye-tracker calibration was adjusted to <1° average accuracy and <0.5° precision before recording (42–44).

We defined the area occupied by the FOPL on each package as an Area of Interest (AOI). To measure sustained attention on the FOPL [conscious perception according to (15)], we recorded the fixation duration (on the AOI in seconds) and the number of fixations (of the AOI). To measure initial orientation [subconscious perception according to (15)], we recorded the time to first fixation on the FOPL (in seconds) from stimulus onset (42, 45).

#### 2.3.1 Stimuli

Our experiment did not involve any conventional manipulation of the stimuli. There was no “distortion” of facts intended to elicit a specific response. However, in our study, packaging was conceived and designed that does not exist in reality. We did not use any existing brands or products in order to control and, as far as possible, eliminate any associated connotations (such as product preferences or purchasing behavior). To make the packaging appear realistic, it was designed in a classic, plain style with only a few distinctive design elements to prevent monotony. Each stimulus in this study was a composite image of a fictional food package front with a nutrition label. The front-of-pack designs depicted fictitious products and were kept simple and low in visual complexity (following guidelines from (46, 47)). We developed a variety of fictional product images for this study. Each package included basic realistic elements (logo, product name, product image, weight), which were held constant in style (see Supplementary Appendix Figures 1–5 for examples). The packages varied by color and by the FOPL displayed (Nutri-Score or Israeli nutrition label). We designed the stimuli to meet several criteria and control for them experimentally. First, following Ketron et al. (48), we included both vice and virtue foods, as these categories might elicit different responses [e.g., focus on taste vs. health, or short-term vs. long-term benefits; (49, 50)]. We selected 5 broad food categories (nuts, cereals, snacks, bars, and burger patties), each represented by two specific products – one relatively healthier and one less healthy. For each product, we also created two versions of the nutrition label (one indicating a more favorable nutritional profile and one indicating a poor profile). Using nutritional criteria from the literature and label specifications, we determined the highest (best) and lowest (worst) Nutri-Score ratings and Israeli nutrition label statuses achievable for each product. This yielded a total of 40 distinct FOP stimuli (5 categories × 2 products × 2 nutritional levels × 2 label types). Table 1 summarizes the products and their label values.

**TABLE 1:** Selected food products and FOP stimuli.

Food category	Food	German Nutri-Score		Israeli warning label	
		Better	Worse	Better	Worse
Nuts	Almonds	A	D	Gl	Fa, Sa
	Cashews	B	C	Gl	Fa, Sa
Cereals	Corn Flakes	A	C	Su	Sa, Su
	Honey Loops	B	D	Su	Fa, Su
Snacks	Cracker	B	C	Sa	Fa, Sa
	Chips	B	D	Sa	Fa, Sa
Bars	Protein Bar	A	D	Fa	Fa, Sa, Su
	Granola Bar	B	E	Fa	Fa, Sa, Su
Burger Patties	Meat	B	C	Fa	Fa, Sa
	Vegan	A	D	Fa	Fa, Sa

“Better” and “Worse” indicate the lowest-risk vs. highest-risk nutritional value for each product under the respective labeling system. Nutri-Score letters A–E are grades from healthiest (A) to least healthy (E). Labels from Israel are denoted as Gl (Green label for healthy product), Fa (“High in fat”), Sa (“High in salt”), Su (“High in sugar”) in the Israeli system.

Background color and salience can influence visual processing (51) and purchase intention (52). Therefore, aside from the burger patties (which had a fixed background), background colors were randomly assigned to the product images (choosing among yellow, red, blue, or green) in order to experimentally control for any color-driven attention effects. Examples of the front-of-pack designs are shown in Supplementary Appendix Figures 1–5.

Ongoing content analysis of front of food package design by our research groups indicates that in both countries most nutrition labels appear at the bottom of the package (53). In addition, we followed the formatting guidelines for each label when designing and placing the food labels on the front of the packaging [see e.g., (54, 55)]. Desk research, as well as evaluations and assessments by nutrition experts on the research team, confirmed that the stimuli were comparable to one another and to real products.

#### 2.3.2 Nutrition label design

**FIGURE 4:**
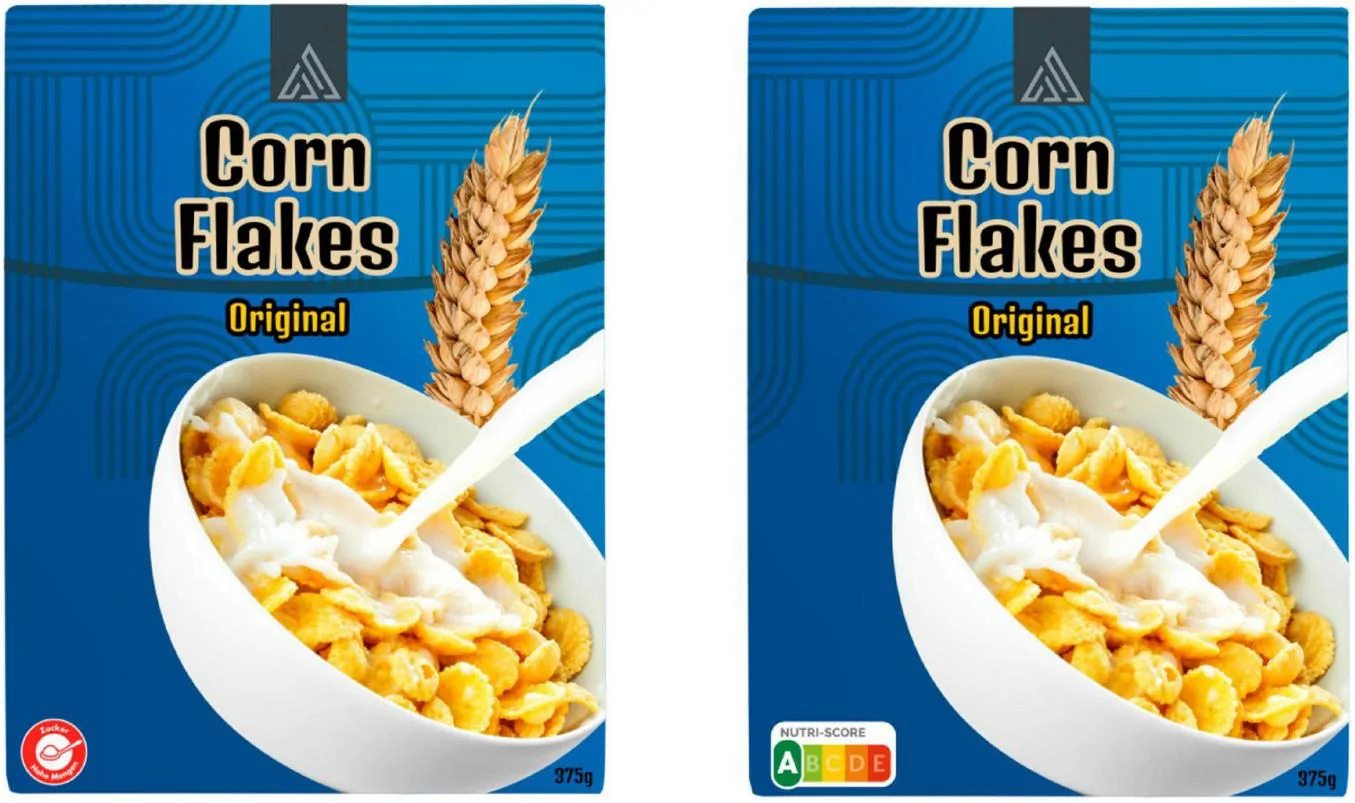
Blue cornflakes package designs with Warning label (left) and Nutri-Score label (right).

In addition to designing the front of packages, we also prepared the nutrition labels for cross-country use. The Nutri-Score graphic was obtained from the official templates (from the German Federal Ministry of Food and Agriculture). For use in Israel, the Nutri-Score label text was translated into Hebrew by the Israeli team, and the graphic was adapted so the label would be understandable in the local language. Similarly, the Israeli labels (original Hebrew versions) were translated into German (and label text replaced with German) to create stimuli for German participants to view unfamiliar Israeli labels. These cross-language adaptations ensured that each participant saw both their country’s familiar label and the foreign label in a comprehensible form (see Supplementary Appendix Figure 6 for the label designs). An example of a product presented with each type of label is shown in Figure 4. The characteristics of the two models (“better” and “worse”) were selected based on comparative desk research. “Better” or “worse” always represent the extremes found and refer to the healthiest and least healthy options that were available in our desk research for each product (see Table 1). For almonds, for example, the “better” option was the natural variety with no additives (Nutri-Score A, Green label), while the “worst” option was roasted and salted almonds (Nutri-Score D, warning label for fat and sodium). For potato chips the “better” option was the natural variety with no additives (Nutri-Score A, Green label), while the “worse” option was high in fat and sodium (Nutri-Score D, warning label for fat and sodium).

#### 2.3.3 Self-report measures

During the second part of the study (after eye-tracking), participants answered questions about the products and labels they had seen. First, they participated in a short experimental survey where they evaluated the healthiness, expected taste, and purchase likelihood of food products that were once presented with labels from the Israeli label system and once with Nutri-Score. Upon completion of the experimental survey, they were asked about their label perception as well as general knowledge and habits.

**Experimental Survey**

The first stage of the survey was an experiment, in which participants were shown images of the FOP stimuli presented in the eye-tracking task (a subset balanced across conditions; see Supplementary Appendix Table 1). The stimuli consisted of four food products presented in two within-subject conditions: with Israeli labels and with Nutri-Score labels, for a total of eight images. The background color was fixed in this subset of the 8 pictures and was not randomized. After viewing each selected product image, participants rated the expected taste, perceived healthiness of the product, and the probability of purchase had they encountered it, all on 7-point Likert scales (from “1 = very low” to “7 = very high”).

**Subjective understanding, knowledge, and attitudes toward FOPL survey**

Participants’ subjective understanding of the label was assessed using items adapted from Fialon et al. (56). Label usage frequency was measured by asking how often they generally pay attention to nutrition labels, on a 7-point scale from “never” to “always”. Label knowledge was tested with four multiple-choice questions about each label system (Nutri-Score and Israeli nutrition labels), to gauge how well participants understood the meaning of each. Finally, general nutrition knowledge was measured using Section 2 of the General Nutrition Knowledge Questionnaire – Revised (GNKQ-R) (57), which assesses understanding of dietary recommendations and nutrient sources. At the end of the survey, standard socio-demographic information was collected, including age, gender, height, weight (used to calculate BMI), education level, household size and income, and any special dietary habits or restrictions.

#### 2.3.4 Data preparation and analysis

Data were collected from a total of 241 participants. Eye-tracking data quality was checked by examining the calibration precision and accuracy for each recording; we applied thresholds of <0.5° for precision and <1° for accuracy. We inspected recordings that barely met these criteria and excluded cases where data quality was too low. In total, 18 participants (9 per country) were excluded due to poor eye-tracking data, yielding a final analyzed sample of 223 participants. We followed standard algorithms for defining eye movement events. In line with Hummel et al. (58), fixations were defined as gaze points that remained within a 1° area for at least 100 ms with a velocity below 30°/s, occurring during the stimulus presentation interval. Using these IV-T filter settings, we extracted for each participant and each stimulus the time to first fixation on the label AOI, the total fixation duration on the AOI, and the number of fixations on the AOI. Additionally, we calculated each participant’s BMI from self-reported height and weight.

For the regression analysis, we fitted linear mixed models using REML estimation and the *nloptwrap* optimizer. Seperate models were estimated for each of the three eye-tracking measures and the three product-evaluation outcomes. Fixed effects included country, labelsystem, and healthiness level, with participant ID and product included as random effects. In addition, we included *age* and *gender* as common sociodemographic covariates, and *Nutrition knowledge* and *frequency of use* given their potential effect on participants attention and response to each type of label. As an example, the model for fixation duration was specified as: *Duration* = *age* + *gender* + *country* + *label system* + *country x label system* + *nutrition knowledge* + *frequency of use* + *healthiness* +*(1—ID)* + *(1—Product)*.

Standardized coefficients were obtained by fitting the model on a standardized version of the dataset. *P*-values were computed using Wald t-distribution approximations.

## 3 Results

### 3.1 Sample characteristics

Table 2 presents a summary comparison of the German and Israeli samples. Overall, the two groups were similar in age, gender distribution, and education level. There were notable differences in employment rate (53% of the German sample vs. 73% of the Israeli sample were employed; χ^2^ = 9.83, *p* = 0.002) and income (34% of Germans reported an income above the national average vs. 18% of Israelis; χ^2^ = 7.37, *p* = 0.025).

**TABLE 2:** Sample characteristics of social demographics.

Characteristic	German sample (*n* = 112)	Israeli sample (*n* = 111)	Overall (*n* = 223)
**Age M (SD)**	27.1 (9.22)	28.4 (6.59)	27.7 (8.03)
Gender n (%)			
Female	67 (60)	55 (50)	122 (55)
Male	45 (40)	55 (50)	100 (45)
Diverse	0 (-)	1 (1)	1 (0)
Education n (%)			
School	58 (52)	40 (36)	98 (44)
College	48 (43)	66 (59)	114 (51)
Practical Training	5 (4)	4 (4)	9 (4)
None, others	1 (1)	1 (1)	2 (1)
Employment** n (%)			
Not employed	53 (47)	30 (27)	83 (37)
Employed	59 (53)	81 (73)	140 (63)
Income* n (%)			
Below average	57 (51)	69 (62)	126 (57)
Average	17 (15)	22 (20)	39 (17)
Above average	38 (34)	20 (18)	58 (26)

*Independent *t*-test was used for age; χ^2^ tests for categorical variables. **p < 0.01, *p < 0.05

### 3.2 Effect of FOPL type and familiarity on visual attention

To evaluate the effects of FOPL type and label familiarity on visual attention, we conducted a series of linear mixed-effects models (LMMs) for each eye-tracking measure (fixation duration, fixation count, time to first fixation) as one dependent variable. Each model included fixed factors for country (Germany vs. Israel), FOPL type (Nutri-Score vs. Warning), and their interaction (country × FOPL, representing familiarity). In addition, we included further fixed factors influencing label perception and evaluation (15, 34, 35) as control variables in the regression models (Table 3). We used random intercepts for participants and for products to account for individual differences and item differences. Number of cases for each regression model, ICC values and R^2^-values are reported for each regression model.

**TABLE 3:** Mixed-model estimates for fixation duration, fixation count, and time to first fixation by FOPL type (fixed effects).

Predictors	Fixation duration				Fixation counts				Time to 1st fixation			
	Estimates	CI	SE	*p*	Estimates	CI	SE	*p*	Estimates	CI	SE	*p*
(Intercept)	0.11	−0.20 to 0.42	0.158	0.501	0.47	−0.59 to 1.53	0.540	0.384	1.05	0.55 to 1.56	0.257	< 0.001
Age	−0.00	−0.01 to 0.00	0.003	0.542	0.00	−0.02 to 0.01	0.009	0.595	−0.00	−0.01 to 0.01	0.004	0.695
Gender (female)	−0.02	−0.11 to 0.06	0.044	0.620	−0.09	−0.38 to 0.20	0.149	0.539	−0.05	−0.19 to 0.09	0.071	0.451
Country (Israel)	0.07	−0.01 to 0.16	0.045	0.100	0.05	−0.25 to 0.35	0.153	0.736	−0.14	−0.28 to 0.01	0.076	0.074
FOPL type (Israeli label system)	0.13	0.07 to 0.19	0.031	**<0.001**	0.55	0.31 to 0.80	0.125	**<0.001**	0.11	0.00 to 0.21	0.054	**0.048**
Country X FOPL	−0.25	−0.29 to −0.21	0.020	**<0.001**	−0.91	−1.04 to −0.78	0.067	**<0.001**	−0.21	−0.31 to −0.11	0.050	**< 0.001**
Nutrition knowledge	0.01	−0.00 to 0.02	0.005	0.160	0.03	−0.00 to 0.07	0.017	0.064	0.01	−0.01 to 0.03	0.008	0.270
Use frequency	0.04	0.02 to 0.07	0.013	**0.001**	0.11	0.02 to 0.19	0.044	**0.014**	0.02	−0.02 to 0.06	0.021	0.311
Health level (lower)	0.11	0.05 to 0.17	0.030	**<0.001**	0.50	0.26 to 0.73	0.120	**<0.001**	0.04	−0.05 to 0.14	0.048	0.361
σ2	0.22				2.50				1.39			
τ00 ID	0.10				1.13				0.24			
τ00 FOP	0.01				0.13				0.02			
ICC	0.33				0.34				0.15			
Marg./Cond. R^2^	0.04 / 0.35				0.05 / 0.37				0.01 / 0.16			

*n* = 8880, n_*ID*_ = 222, n_*FOP*_ = 40, CI = 95% Confidence interval, SE = Standard Error, Marg. R^2^ = Marginal R^2^, Cond. R^2^ = Conditional R^2^. Bold values indicate significant (*p* < 0.05) estimates.

**FIGURE 5:**
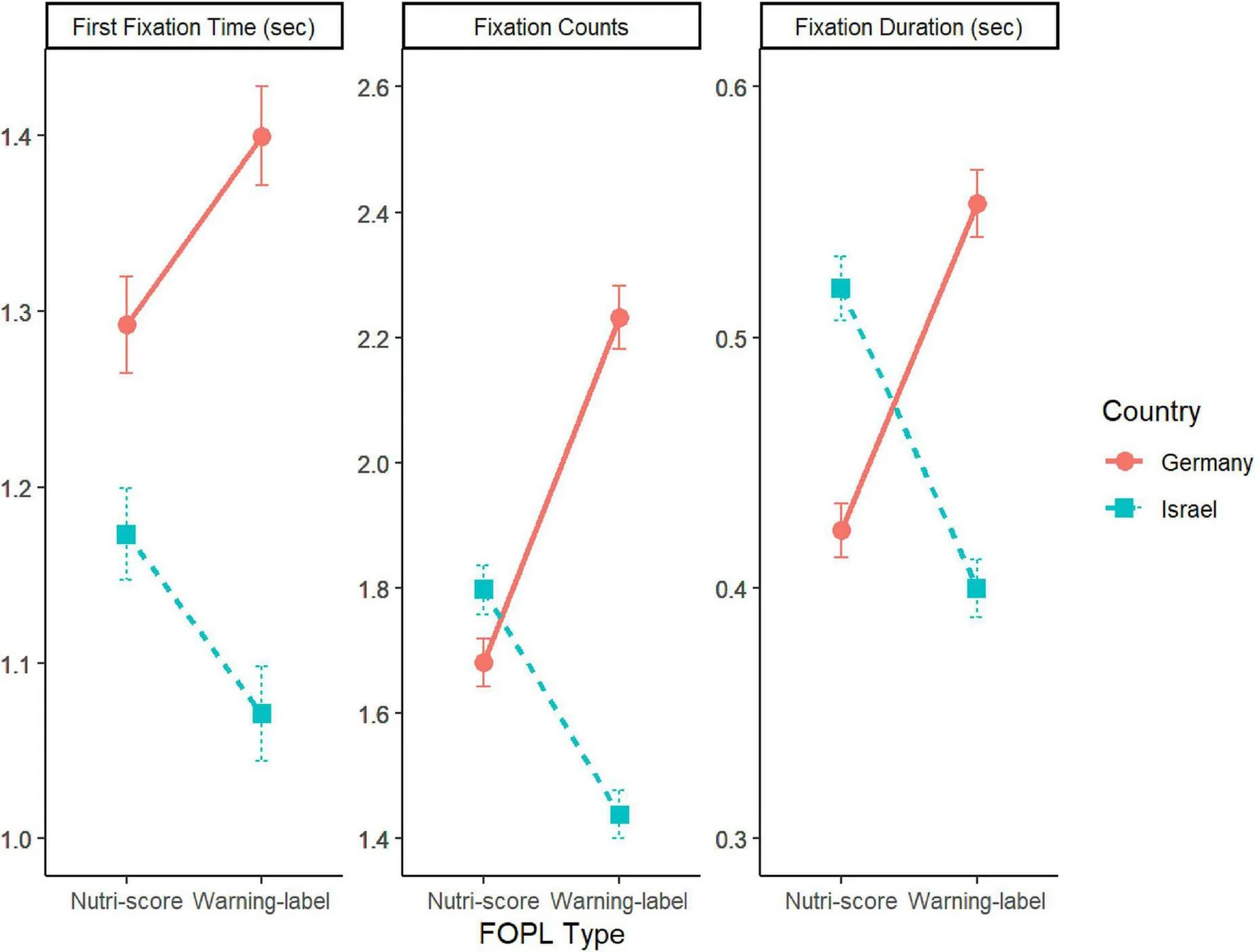
Mean fixation duration, fixation count, and time to first fixation by FOPL type and country (error bars indicate ± 1 SE).

The results of the linear mixed models regression analysis (Table 3) reveal a significant effect of FOPL type on all visual attention measures, as well as a significant familiarity interaction also on all visual attention measures. German participants fixated longer (Model 1) and more frequently (Model 2) on the Israeli label system than on the Nutri-Score, whereas the opposite was true for Israeli participants (Figure 5). In addition, participants in Germany fixated on the Nutri-Score sooner (shorter time to first fixation, Model 3), while Israeli participants fixated on the Israeli labels sooner. These patterns indicate a strong familiarity effect on visual attention: In both countries, people spent more time looking at the unfamiliar label (the Israeli labels for Germans, the Nutri-Score for Israelis) than at their familiar label. Notably, in both countries the familiar FOPL was detected faster (shorter time to first fixation) than the unfamiliar label.

The frequency of use and the health level of the food are additional influencing factors, particularly for the measurements of maintained attention. The duration and the number of fixations increase with increasing use of labels in everyday life and a depicted unhealthier product. Interestingly, for initial attention, measured as time to first fixation, we only find an influence of label type and familiarity. In other words, both influences are the main drivers of initial orientation to a label in the first milliseconds.

### 3.3 Effect of FOPL type on perceived taste, healthiness, and willingness to purchase

For the product evaluation outcomes, we again fit three linear mixed-effects models predicting participants’ ratings of expected taste, perceived healthiness, and purchase likelihood. Each model included fixed effects for country (Germany vs. Israel), FOPL type on the product (Nutri-Score vs. Israeli label system), and their interaction, plus the same set of covariates (age, gender, nutrition knowledge, label use frequency, product health level) with random intercepts for participant and product.

Table 4 summarizes the regression results for these three dependent variables. For taste ratings the explanatory power was modest. There was no significant main effect of label type on taste expectation or the familiarity of the labels. Age had a small negative effect, indicating that older participants tended to expect slightly worse taste. Frequent consumption of the product category was positively associated with taste ratings, suggesting that familiarity with a product leads to a better expected taste.

**TABLE 4:** Mixed-model estimates for product evaluations: taste, healthiness, and purchase likelihood (fixed effects).

Predictors	Taste				Health				Purchase			
	Estimates	CI	SE	*p*	Estimates	CI	SE	*p*	Estimates	CI	SE	*p*
(Intercept)	5.53	4.39 to 6.68	0.586	<0.001	5.42	4.11 to 6.73	0.668	**<0.001**	4.54	3.19 to 5.90	0.691	**<0.001**
Age	−0.02	−0.03 to −0.00	0.007	**0.015**	−0.01	−0.03 to −0.00	0.006	**0.018**	−0.00	−0.02 to 0.01	0.008	0.746
Gender (female)	−0.04	−0.27 to 0.19	0.119	0.739	−0.10	−0.29 to 0.09	0.098	0.306	0.20	−0.06 to 0.46	0.132	0.128
Country (Israel)	−0.05	−0.32 to 0.22	0.137	0.700	0.66	0.44 to 0.87	0.110	**<0.001**	0.54	0.25 to 0.84	0.151	**<0.001**
FOPL type (Israeli label system)	−0.07	−0.99 to 0.86	0.470	0.887	−0.48	−1.77 to 0.81	0.659	0.468	−0.29	−1.45 to 0.87	0.591	0.621
Country X FOPL	0.12	−0.15 to 0.38	0.134	0.383	−0.16	−0.36 to 0.03	0.098	0.098	0.16	−0.13 to 0.44	0.145	0.281
Nutrition knowledge	−0.02	−0.05 to 0.01	0.014	0.197	−0.03	−0.05 to −0.01	0.012	**0.010**	−0.03	−0.06 to −0.00	0.015	**0.026**
Use frequency	0.07	0.00 to 0.14	0.035	**0.043**	0.06	0.00 to 0.12	0.029	**0.043**	0.10	0.02 to 0.17	0.039	**0.013**
Health level (lower)	−0.34	−1.25 to 0.58	0.465	0.468	−1.98	−3.27 to −0.69	0.657	**0.003**	−0.84	−1.99 to 0.31	0.587	0.152
σ2	2.00				1.08				2.32			
τ00 ID	0.52				0.39				0.64			
τ00 FOP	0.42				0.86				0.68			
ICC	0.32				0.54				0.36			
Marg./Cond. R^2^	0.03 / 0.34				0.34 / 0.69				0.09 / 0.42			

*n* = 1776, n_*ID*_ = 222, n_*FOP*_ = 8, CI = 95% Confidence interval, SE = Standard Error, Marg. R^2^ = Marginal R^2^, Cond. R^2^ = Conditional R^2^. Bold values indicate significant (*p* < 0.05) estimates.

For healthiness ratings, the regression model had the highest explanatory power. Neither the label alone nor the familiarity of the label (in the form of interaction) had a significant influence on the evaluation of the healthiness of the product. Instead, the country was a significant predictor: Israeli participants gave products higher health scores than German participants, indicating cultural or contextual differences in health perceptions. Nutrition knowledge had a significant negative effect on healthiness ratings, suggesting that more knowledgeable consumers were more critical in their health evaluations. Label use frequency correlated positively with health ratings (those who often use labels tended to rate products as healthier). Importantly, product health level (whether the item was the “less healthy” version) had a strong negative effect on the health rating, confirming that our stimuli manipulation of nutritional quality was reflected in participants’ perceptions. The FOPL type (warning vs. Nutri-Score) did not show a significant main effect on health ratings overall.

For purchase likelihood, neither the label alone nor the familiarity of the label had a significant effect. Instead, again country had a positive influence on purchase intention, meaning Israeli participants reported a higher willingness to buy the products than German participants, possibly reflecting generally higher baseline purchase likelihood or cultural differences. Frequent label users had significantly higher purchase intentions, suggesting those who pay more attention to labels tend to be more willing to purchase, perhaps due to trust or interest in the information. FOPL type did not significantly affect purchase likelihood overall, nor did the interaction with country.

**FIGURE 6:**
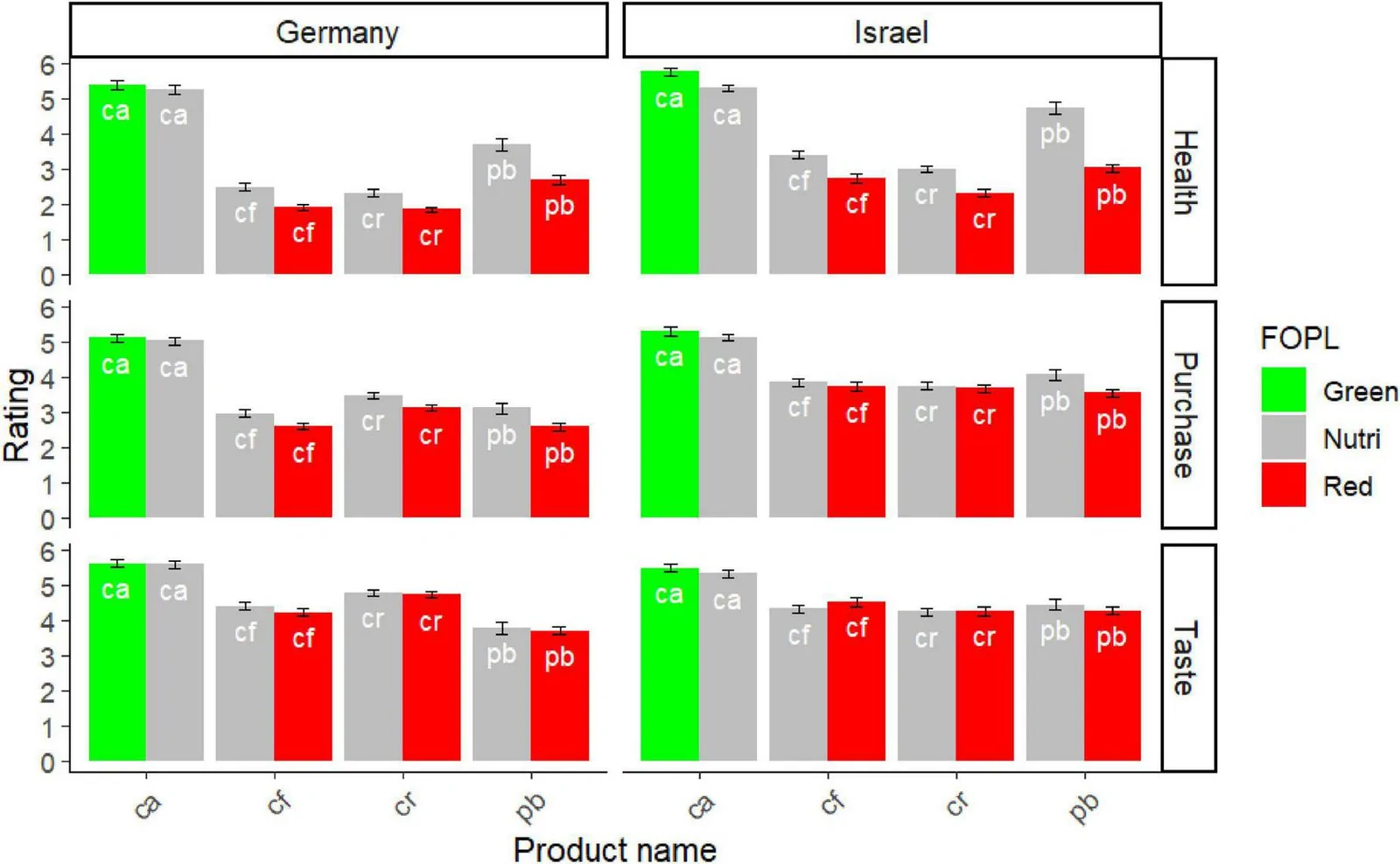
Mean consumer ratings of perceived healthiness, taste, and purchase intention for each product, by FOPL type (warning vs. Nutri-Score) and country. “ca” = cashews, “cf” = cornflakes, “cr” = crackers, “pb” = protein bar (the four food products examined in the experimental survey).

It is notable that no strong main effect of label format was found on taste, health, or purchase ratings in the aggregated analysis. This could result from the fact that the Israeli FOPL in this analysis included both the warning red labels on three products but also a positive green label on the fourth product (cashew). Therefore, we continued by running exploratory analyses that separated the two types of labeling. This analysis revealed nuanced differences when looking at specific products and label conditions Figure 6 illustrates the mean perceived health, taste, and purchase intention for each product under the two label types (warning vs. Nutri-Score) in each country. Overall, the patterns of label influence were similar in Germany and Israel, and somewhat consistent across outcome measures. Nevertheless, Nutri-Score labels tended to produce more moderate evaluations compared to the Israeli labels – that is, Nutri-Score did not influence perceptions as strongly, either positively or negatively, as the presence or absence of warning labels did. For example, Nutri-Score yielded a lower health rating than the green positive label for a healthy item like raw cashews (since cashews with a green “healthy” label were rated very high), but Nutri-Score resulted in a higher health rating than the red “High in” labels for less healthy items such as cornflakes, crackers, or protein bars (which were strongly marked by red warnings). A similar effect was observed for expected taste and purchase intentions, where Israeli labels tended to noticeably lower ratings for some items, as compared to the Nutri-Score labels.

To get basic insights into the attitudes and knowledge regarding FOPLs we assessed participants’ self-reported understanding and opinions of each label type after they had experienced both. Table 5 summarizes the results for label-specific knowledge, and several 7-point scale attitude questions about each system’s informativeness, trustworthiness, comprehensibility, and helpfulness for making healthy choices, as well as reported frequency of label use in daily life.

**TABLE 5:** Comparison of attitudes toward Nutri-Score vs. Israeli label system (overall sample *N* = 223, Germany *n* = 112, Israel *n* = 111).

Attitudes and characteristics	Sample *n* = 223	Germany *n* = 112	Israel *n* = 111	*t* ^iii^	*d*
Label Knowledgei M ± SD					
Nutri-Score	3.0 ± 0.72	3.2 ± 0.68	2.8 ± 0.72	3.82***	0.51
Israeli labels	3.7 ± 0.56	3.7 ± 0.57	3.7 ± 0.56	0.16	0.02
*t* ^iv^	12.34***	7.32***	10.31***		
Cohen’s d	0.83	0.69	0.98		
Label evaluation M ± SD					
Information about nutritional quality					
Nutri-Score	4.3 ± 1.79	4.1 ± 1.80	4.5 ± 1.77	−1.78	−0.24
Israeli labels	4.7 ± 1.57	4.9 ± 1.36	4.6 ± 1.75	1.08	0.15
*t*	3.22**	4.07***	0.47		
Cohen’s d	0.22	0.39	0.04		
Trustworthiness					
Nutri-Score	4.4 ± 1.67	4.1 ± 1.68	4.6 ± 1.63	−2.08*	−0.28
Israeli labels	4.7 ± 1.57	4.4 ± 1.52	5.0 ± 1.55	−3.22**	−0.43
*T*	2.85**	1.23	3.21**		
Cohen’s d	0.19	0.12	0.31		
Comprehensibility					
Nutri-Score	4.6 ± 2.05	4.6 ± 2.04	4.5 ± 2.07	0.15	0.02
Israeli labels	6.0 ± 1.25	5.8 ± 1.40	6.2 ± 1.06	−2.27*	−0.30
*t*	9.76***	5.61***	8.41***		
Cohen’s d	0.65	0.53	0.80		
Healthful choice					
Nutri-Score	4.1 ± 1.91	3.7 ± 1.84	4.4 ± 1.93	−2.52*	−0.34
Israeli labels	4.7 ± 1.67	4.6 ± 1.64	4.8 ± 1.70	−0.79	−0.11
*t*	5.36 ***	4.74***	2.73**		
Cohen’s d	0.36	0.45	0.26		
**Label usage *M* ± *SD***	3.6 ± 1.70	3.4 ± 1.55	3.9 ± 1.82	−2.16*	−0.29

*i* Label Knowledge score: sum of correct answers to four knowledge questions per label system (max = 4). *ii* Label Evaluation rated on 7-point Likert scale (1 = strongly disagree, 7 = strongly agree) for Nutri-Score (NutS) and Warning Label (WL) systems. Statements: “The Nutri-Score / Warning Label **informs me about** the nutritional quality of food,” “… **is trustworthy**,” “… **is easy to understand**,” “… **helps me to choose** healthy products.” *iii* Between-country difference (independent t-test for Germany vs. Israel). *iv* Within-subject difference (paired *t*-test comparing Nutri-Score vs. Israeli Label ratings within participants). Significance: ***p < 0.001; **p < 0.01; *p < 0.05.

Analysis (Table 5) shows that participants’ label-specific knowledge was higher for the the Israeli label system than for the Nutri-Score. This suggests that the Israeli label system was more straightforward to understand or remember compared to the Nutri-Score’s grading system, even for those less familiar with it. In terms of subjective label evaluations, the Israeli labels were rated more favorably on key aspects. Participants in both countries found the Israeli labels easier to understand than the Nutri-Score. They also agreed more strongly that Israeli labels help in choosing healthy products. There were no differences in perceived informativeness, though German participants found the Israeli label system significantly more informative than the Nutri-Score. Trustworthiness ratings showed a nuanced pattern: Israelis perceived the Israeli labels as more trustworthy than Nutri-Score, while Germans actually gave a slight edge to Nutri-Score on trust. This led to no significant overall difference in trust ratings between labels. Reported label usage frequency differed by country: participants in Israel reported using nutrition labels more frequently on average than those in Germany. This suggests that the presence of the mandatory Israeli label system in Israel might be associated with greater habitual use of labels, though other cultural factors could also contribute. In summary, participants from both countries demonstrated higher knowledge and better subjective comprehension of the Israeli label system compared to Nutri-Score. They also generally perceived the Israeli labels as more helpful for making healthy choices. However, perceptions of trustworthiness and informativeness were mixed, with some country-specific differences, as German participants found warnings more informative, while Israeli participants found them more trustworthy.

## 4 Discussion

This study provides novel evidence on how the common FOPL scheme types, evaluative summary (Nutri-Score) vs. nutrient specific (warning) capture consumers attention, and how they are visually perceived and evaluated by people who are familiar or unfamiliar with them. The binational experiment allowed us to study the reactions to each label scheme, while controlling for familiarity, as well as specifically study its effect on consumer reactions to the label, from attention to judgment and choice and to attitudes. To the best of our knowledge, this is the first study to do so. In addition, this is the first research that assesses all stages of the intended (theoretical) consumer reaction to the FOPL: from attention to product evaluation, and to knowledge and attitudes toward each scheme. As such, the current study provides a comprehensive comparison of the effectiveness of main FOPL schemes on consumer reaction.

The label system significantly influences visual attention, particularly with respect to fixation duration and number of fixations which stand for maintained attention (29) or conscious perception (15). This shows that FOPL design plays a central role in information processing. Importantly, familiarity emerged as a key factor in visual perception. We found that participants fixated more times, and for longer period of time on the FOPL format that was unfamiliar to them, a result that seems consistent with previous studies studying familiar vs. unfamiliar foods (59). Participants fixate the familiar label of their home country more quickly, which could indicate unconscious perception and orientation in the early stages of perception. This observation suggests the role of repeated exposure and learning: people experienced with a given label may process it more efficiently, whereas a new label draws extended attention, possibly as consumers take time to interpret it. Such familiarity effects have been suggested in the literature (25), and our results confirm that introducing a new label will likely spark an initial increase in attention. The current experimental setup does not allow to determine whether the observed effect stems from the novelty of the foreign label (60) or from the familiarity of the local one. This distinction warrants further investigation in future studies. Nevertheless, the findings indicating that familiar FOPLs are attended for shorter durations and detected earlier than unfamiliar ones, suggest a distinction that may be relevant for understanding the temporal development of exposure and familiarity. They also suggest that consumers’ processing of FOPLs becomes more efficient over time, as labels are spotted more quickly and may require less cognitive processing.

Notably, while the models for total fixation duration and count had high explanatory power, the model for time to first fixation had relatively low explanatory power, suggesting that other factors (e.g., package design elements or individual differences in scanning patterns) also play a role in how quickly a person initially notices a label. These results have implications for label design: new labels should be designed for eye-catching to capture attention, whereas familiar labels might need periodic redesign or complementary education to encourage deeper processing (61). We can confirm the importance of signal colors (16, 24, 62) based on our findings. In particular, the Israeli label system with its combination of signal colors with clear warning messages has a significant effect. In future studies, the third element (the distinctive shape of the label, as in the case of black hexagon labels) could also be included in a comparison to obtain even more information about the effectiveness regarding the warning labels (62). Overall, our eye-tracking findings provide valuable evidence that label familiarity and salience can markedly affect the visual attention to nutrition information on packaging.

Beyond visual attention, our results show that age, cultural context, nutrition knowledge, and label use habits all play a role in product evaluation. People with higher general nutrition knowledge were more critical in their ratings – they gave lower healthfulness scores and lower purchase likelihood, which might indicate greater skepticism toward perceived health claims or an ingrained caution [see e.g., (63)]. We also found that label usage influences perception: participants who frequently use nutrition labels in real life rated products as healthier and were more willing to buy them (perhaps reflecting that health-conscious individuals both use labels more and view products more favorably when they have positive nutritional attributes). Country of origin had a strong influence on health ratings and purchase probability – Israeli participants tended to rate products healthier and showed higher purchase intent than Germans, across all displayed products. This could point to cultural differences in rating tendencies or in baseline exposure to the product types, and it suggests that local context matters when interpreting absolute rating scales in such experiments.

One intriguing outcome was that the presence of a positive green label (in the Israeli system) on a product seemed to elevate healthier product perception even than a Nutri-Score “A”. In the Israeli sample, products with the green label were rated much healthier than those without, indicating a halo effect of the positive endorsement label. Even in comparisons with Nutri-Score, which also uses green for its top grade, the explicitly positive framing of “this is a healthy choice” might lead to a stronger effect than a gradated score. This aligns with other studies suggesting that positively framed labels can create a more favorable health perception of a product (64).

We also found that products with a poor nutritional profile were rated as healthier when bearing a Nutri-Score than when marked with a red warning – suggesting a “health halo” effect from the Nutri-Score, relative to the more explicit negative warning. Conversely, Israeli labels induced more critical health evaluations and lower purchase intentions for the same products, indicating their potential to reduce the appeal of less healthy items. These findings seem consistent with a recent study that compared Nutri-score with the Chilean (black octagon) warning labels in a quasi-experimental survey, to find that fewer people identified products as low nutritional quality when they were labeled with the Nutri-score than unlabeled products, while warnings increased their proportion. Interestingly, these findings were particularly relevant for products with a “health halo” such as yogurt (65).

Participants also reported that the Israeli label system was easier to understand and more useful than Nutri-Score, regardless of familiarity, supporting the notion that simple nutrient-specific warnings are intuitively grasped.

Another notable finding concerns consumers’ general attitudes toward the two labels. Both German and Israeli participants expressed more positive perceptions of the Israeli label system than of the Nutri-Score (Table 5). Although earlier studies have documented favorable attitudes toward the Nutri-Score [e.g., (66, 67)], the current results do not contradict these findings. The Nutri-Score was also rated positively, but the Israeli labels received even higher evaluations. The current results deviate, however, from previous studies that showed better objective understanding of the Nutri-Score over warnings (16, 18, 24). Several methodological differences may account for this discrepancy. For example, Engell et al. examined the South American black-heptagon warnings, which differ visually from the Israeli red-circle warnings. More importantly, their task involved rating (and choosing between) three products presented simultaneously, whereas the current study presented a single product at a time. In a comparative setting, Nutri-Score may facilitate easier differentiation between products as it provides a graded rating rather than a binary warning or a positive endorsement. However, when evaluating a single product, the more specific information conveyed by warnings may be more informative than a general score. This proposed interaction between label design and decision context in shaping consumers’ understanding and use of FOPL warrants a closer examination in future research.

The stronger performance of the Israeli label system in capturing attention and conveying an unambiguous health warning, suggests that a “high in” warning system could be more effective in steering consumers away from products rich in sugar, salt, or saturated fat. This resonates with the approach and recommendations of health authorities in other regions (e.g., PAHO’s endorsement of warning labels as effective tools). In theory, Nutri-Score’s graded summary provides a nuanced view of overall nutritional quality and enjoys broad support from consumer and health organizations in Europe. Advocates of Nutri-Score argue that it helps consumers compare options within a category to make healthier swaps. Our findings indicate that such a graded label may make unhealthy products seem somewhat more acceptable (relative to a warning), which is a double-edged sword: it may encourage reformulation and healthier choices within categories, but it might also diminish the deterrent impact for products that are still nutritionally poor. If the primary goal is to discourage consumption of foods high in critical nutrients, nutrient-specific warnings might yield stronger results. If the goal is to gradually improve diets by encouraging better choices across the spectrum, a summary indicator like Nutri-Score may be a better fit, provided the policy is mandatory and the formula used does in fact result in an industry producing healthier food options within a category.

**Strengths and limitations**

Our study has several notable strengths, including the incorporation of objective eye-tracking measures and a unique cross-country design to examine real-world implemented labels and to enable the examination of familiarity. Our findings are consistent with prior research on FOPL [e.g., (8, 61, 68)] and extend that body of work by incorporating an eye-tracking component to capture visual attention, which is a fundamental first step in how labels might influence consumer behavior, as their design is intended to make them easier to spot and comprehend. By conducting the experiment in two countries that implemented different FOPL systems, we also directly examined the role of familiarity on the one hand and context on the other (e.g., country characteristics) in label effectiveness.

However, we acknowledge certain limitations. First, the experiment was conducted in controlled lab settings and focused on a set of product stimuli, which do not capture the full complexity of grocery shopping environments. While our eye-tracking experiment allowed for a fully controlled setting, a necessary condition for internal validity, we did not intend to mimic real-world shopping conditions. Real-world factors like price, time pressure, shelf clutter, and marketing influences were not present. Participants knew they were in an experiment and only viewed products without actual purchasing consequences. Online experiments on FOPL, such as Hammond et al. (25) study, have the advantage of large reach and low cost, but they often cannot capture real attention shown by visual attention and may suffer from reduced control. Our lab-based approach ensured a controlled environment and high-quality eye-tracking data. Future research should strive to combine these approaches, perhaps using mobile eye-tracking in actual shopping environments. Our findings can be seen as a reference point for future studies conducted in more realistic settings. It can therefore be assumed that, under more realistic conditions, the type of label can influence perception and, consequently, consumer decision-making. In the hierarchy of consumer decision-making, perception through visual attention of labels plays a decisive role. Under isolated conditions, we were able to demonstrate that the type of label affects visual attention. In more realistic settings, it remains to be seen how significant these isolated effects are, whether they still play a major role, and what competing influences exist alongside them. We have designed the products to address the influence of existing product preferences, likes, attitudes, and purchasing behavior. In designing the packaging and positioning the labels, we drew on desk research into existing products and also took into account the guidelines and manuals for label placement. Finally, as an interdisciplinary team of experts, we assessed whether the designs and labels appeared comparable to realistic alternatives. Even though the preliminary research was extensive and the design results were reviewed by a team of experts, the lack of a comprehensive pretest represents a shortcoming that must be clearly identified as a limitation of our study.

Second, we were guided by earlier studies and compared different types of labels (16, 24, 25, 28). These labels are intended to have different effects. While warning labels are designed to discourage consumption, graded labels are primarily intended to inform individuals. It is possible that the mechanism behind the “warning effect” is of a more fundamental nature and does not necessarily depend on specific conditions, such as a consumer seeking for information. Follow-up studies could incorporate these assumptions into their examination of how different labels work.

Third, while our sample spanned two countries with distinct labeling schemes, both countries are developed economies with health-conscious populations. Therefore, the results may not be fully transferable to populations in other countries with identical labeling systems. Similarly, our samples do not allow for generalizations about other subpopulations within these countries that have different sociodemographic characteristics. Future studies should include more diverse, larger, and, where appropriate, population-representative samples, possibly from additional countries or with different labeling systems, to confirm whether these effects are generally applicable.

Another limitation is the set of included control variables. We focused on some key moderators like age, gender, nutrition knowledge, and label use frequency. While some of these showed significant effects, the list is not exhaustive. Due to the pre-registration and the risk of overfitting the estimation models, it was impractical to include all of the potential variables that we had initially considered adjusting for in the regressions. Instead, we used them only to determine whether the samples differed in terms of these characteristics. Other factors such as general health consciousness, time pressure, hunger state, or color vision differences, which might also affect responses to labels were not explicitly controlled here. Further studies should expand or adapt the range of control variables to see if the observed effects remain robust.

Although the Hebrew and German versions of the labels were carefully designed to be identical for all intents and purposes, the German and Hebrew versions necessarily have subtle differences in the length of the text. Therefore, however unlikely, we cannot rule out the possibility that this might have a small differential effect on the perception of the labels by each population.

## 5 Conclusions

This study provides novel insights into the attention, perception and evaluation of foods with prominent but different front-of-pack labeling systems in Israel (warnings and green label system) and Germany (Nutri-Score). While many other studies already showed that simple and visually salient warning labels are effective and easy to understand (24, 69) our study adds to the body of literature in several ways. First, we present cross-national experimental evidence in which a nutrition specific warning label is directly compared to a summary interpretative label, the Nutri-Score under identical task and stimulus conditions, rather than relying on separate national case studies. Second, by combining eye-tracking data with self-reported perceptions and purchase intentions, we followed the multi method approach suggested by Grunert and Wills (15) and went beyond stated preferences and showed how different FOPLs might influence and guide visual attention at the point of sale or choice. Third, we examined how several influences such as familiarity, the use of labels, nutrition knowledge and age influence the effectiveness in the comparison between warning labels or green label and the Nutri-Score. Together our study refines and enhances the conclusions of existing studies by showing that not only “simple” warnings are preferred, but also for whom and how they exert their effects in real time decision processes across different regulatory and cultural context.

We found support for the hypothesized influence of warning labels with products labeled by explicit “High in” warnings being generally viewed as less favorably in terms of health and purchase intention. At the same time, our eye-tracking comparison provides evidence on how visual attention differs between two FOPL systems in a binational context, confirming the influence of familiarity. These patterns are consistent with reports suggesting that habitual exposure can lead to faster but less effortful processing. But it could also suggest that after consumers become familiar with a label, they may only need to spot it (as they already know its meaning) and might actually be better in spotting it and processing it more effectively. We also found that other factors – such as routine use of label systems in daily life, individual nutrition knowledge, and age – play a role in how FOPLs are perceived and utilized. Simply implementing a label is not a panacea; how well people understand and incorporate the label into their decision-making is crucial. Our findings suggest that warning labels, with their straightforward message, may be more intuitively understood and impactful, whereas Nutri-Score provides nuanced information but may require greater consumer education for optimal use.

These findings come at a critical time for FOPL policy development in both Europe and globally. In the EU, a decision on a harmonized front-of-pack nutrition label is highly anticipated as part of the Farm to Fork initiative, yet disagreements over the preferred format have thus far stalled progress. Our results provide empirical data that may be useful for future comparative studies on FOPL formats. The strong impact of the warning label in drawing attention and reducing the perceived healthfulness of unhealthy products suggests that a nutrient-specific warning system could influence consumers’ perception and evaluation of products at the point of purchase – essentially by clearly signaling which products consumers should treat with caution. This is conceptually similar to the results from Chile and Mexico, where “high in” labels were associated with changes in reported purchases.

In conclusion, introducing FOPL schemes can be beneficial for guiding healthier choices, but their success depends on both design and integration into consumers’ daily lives. Policymakers and health authorities should consider not only which label conveys nutritional information most effectively, but also how familiarity and education influence the label’s effectiveness. Enhancing public understanding and acceptance of the labels through education campaigns will be necessary to maximize their impact. A label system must attract attention, be easily understood, and maintain its effectiveness even after it becomes familiar to consumers. By addressing both the *attention-grabbing* and *comprehension* aspects of FOPL, such interventions stand a better chance of improving dietary choices and public health in the long run.

## Data Availability

The raw data supporting the conclusions of this article will be made available by the authors, without undue reservation.
