# Supplementary material for: Warning and green labels versus Nutri-Score: visual attention and product evaluation in a binational eye-tracking study
Source: Front Nutr. 2026 Aug 27;13:1826542. doi: 10.3389/fnut.2026.1826542 (PMC13560643; doi:10.3389/fnut.2026.1826542)
Supplement: Supplementary file 1 [file Table_1.docx]

**Figure A1:** Front of Pack designs – Nuts

**Figure A2:** Front of Pack designs – Cereals

**Figure A3:** Front of Pack designs – Snacks

**Figure A4:** Front of Pack designs – Bars

**Figure A5:** Front of Pack designs – Burger Patties

**Figure A6:** Label designs

**Table A1:** FOP and FOPL in Product-Evaluation-Task

| Food | Color | German Nutri-Score | Israeli Warning Label |
| --- | --- | --- | --- |
| Cashews | **yellow** | B | Gl |
| Corn Flakes | **blue** | C | Sa, Su |
| Cracker | **green** | C | Fa, Sa |
| Protein Bar | **red** | A | Fa |

***Note:*** *The letters A – E show the values of the German Nutri-Score system. Gl (Green label), Fa (Warning label for high amounts of fat), Sa (Warning label for high amounts of salt) and Su (Warning label for high amounts of sugar) represent the Israeli Nutrition Label System.*
